# Prevalence of Dental Anxiety and the Influence of Various Factors in Children Aged 12 to 18 Years in Zagreb, Croatia

**DOI:** 10.3390/children13101384

**Published:** 2026-10-12

**Authors:** Lucija Koturić Čabraja, Walter Dukić, Matea Lapaš Barišić

**Affiliations:** 1 Private Dental Practice Zagreb, 10000 Zagreb, Croatia; koturiclucija@gmail.com; 2 School of Dental Medicine, University of Zagreb, Gunduliućeva 5, 10000 Zagreb, Croatia; 3 Faculty of Dental Medicine and Health, Josip Juraj Strossmayer University of Osijek, Crkvena 21, 31000 Osijek, Croatia; ahmlb7@gmail.com

**Keywords:** test anxiety scale, dental anxiety, dental fear, children, socioeconomic factors, dietary habits, oral hygiene, cross-sectional studies

## Abstract

**Highlights:**

**What are the main findings?**

• Dental anxiety decreases over time.
• Children aged 12–18 years have mild to moderate levels of dental anxiety.

**What are the implications of the main findings?**

• There is no difference in dental anxiety levels related to gender or socioeconomic status.
• Financial possibilities, type of dental service, and experience with the dentist may influence the level of dental anxiety.
• Low-cariogenic diet may be associated with a greater reduction in dental anxiety levels after the clinical examination.

**Abstract:**

**Background/Objectives**: Children who experience high levels of dental anxiety tend to exhibit poor cooperation during dental visits, which compromises treatment outcomes and leads to a vicious cycle with poor oral health and caries prevention outcomes. The purpose of this study is to analyze the prevalence of dental anxiety in a sample of clinical subjects with children and possible correlations with other factors, as well as the longitudinal nature of anxiety. **Methods**: This cross-sectional and longitudinal study was conducted on a sample of 150 children aged 12 to 18 years in the city of Zagreb and Zagreb County. The MDAS (Modified Dental Anxiety Scale) was used to collect data related to dental anxiety at three time intervals: before the clinical examination (T1), after the clinical examination (T2), and after a period of 3 months (T3). **Results**: Significant differences between the MDAS before and after the clinical examination were found, suggesting that dental anxiety decreased in most children (*p* < 0.001). The MDAS test results were 9.79 and 8.03 for the T1 and T2 periods, respectively, indicating mild anxiety. There are statistically significant differences between individual time points T1, T2, and T3 (*p* < 0.001), and these differences are significant between T1 and T2 (*p* = 0.045) and between T1 and T3 (*p* = 0.012), while between T2 and T3, the differences are not statistically significant (*p* = 0.616). At T1, children mostly exhibited mild dental anxiety (55.3%), followed by moderate (41.3%) and severe dental anxiety (3.3%). At T2, children mostly exhibited mild dental anxiety (77.2%), followed by moderate anxiety (22.8%) and no severe dental anxiety. Financial influence on the use of dental services (*p* = 0.014), the type of dental service [public/private/semi-private] (*p* = 0.008), and experience with the dentist (*p* = 0.004) were statistically significant factors. The initial MDAS and cariogenic diet scores (*p* < 0.012) have a negative correlation with the change in the MDAS score; greater fear before the clinical examination (T1) is associated with a greater reduction in fear after the clinical examination (T2). A low-cariogenic diet is associated with a greater reduction in fear after the clinical examination (T2). **Conclusions**: Most of the children in this study had mild to moderate anxiety, which decreased after a visit to the dentist. Financial influence on the use of dental services, the type of dental service, and experience with the dentist may be associated with dental anxiety. No statistically significant difference was found between dental anxiety and gender or socioeconomic status. Furthermore, a cariogenic diet may also be associated with higher dental anxiety. Better oral hygiene and oral status, higher socioeconomic status, and a low-cariogenic diet may influence the dental anxiety levels in children.

## 1. Introduction

Dental anxiety is a state of fear that something terrible will occur in connection with dental treatment and it is usually associated with a feeling of loss of control. In contrast, dental fear is a normal emotional reaction to one or more specific threatening stimuli in a dental situation [1], and dental phobia refers to a severe type of dental anxiety, characterized by marked and persistent anxiety related to clearly identifiable situations or objects such as dental drilling, local anesthetic injections, or the dental environment in general [1]. The differential diagnoses of these different types of dental anxiety are subjective, and no clear demarcation has been presented in the literature. Dental phobia is often associated with vasovagal reactions such as increased heart rate due to sympathetic activation, in contrast to dental anxiety or fear. Blood, injection, and injury phobias are associated with initial tachycardia, followed by bradycardia, hypotension, dizziness, and nausea [2]. In general, dental fear is a normal reaction in children and adults to an unfamiliar situation. Mild fear and anxiety are expected experiences, consistent with normal development, but they can become worrisome and may require treatment when the fear or anxiety is disproportionate to the actual threat [3]. Studies have shown that fear of dental treatment can be formed during adolescence, leading to the avoidance of regular dental treatments. Fear can reduce a child’s cooperation and lead to delays in dental treatment. Children who experience high levels of dental anxiety often show poor cooperation during dental visits, which compromises treatment outcomes and increases the stress of dental staff. This can result in more invasive procedures later on, such as deep-caries treatments, tooth extractions, surgical interventions requiring antibiotics, and even hospitalization. As a result, dental caries and abscesses are more likely to occur due to poor and irregular preventive and therapeutic practices, and in turn, such poor oral health leads to increased anxiety and fear, thus creating a vicious cycle [4,5]. This vicious cycle also occurs in adults; it can be considered to have begun in childhood and then to have carried on through subsequent age groups [6,7,8,9]. Numerous factors likely influence the development of dental anxiety, such as past negative dental experiences; the experiences of other patients and their attitudes; comments on social media; pain and other clinical symptoms; the dental office environment, particularly the appearance of the waiting room; the sounds of drills; and the smell of dental materials [10,11,12]. It has also been proven that anxiety is directly influenced by parents on children and that there are differences in the influence of anxiety related to the gender of parents and children, and in the influence of mothers on the child regarding anxiety [13,14,15,16]. Other possible causes of dental anxiety include the level of oral hygiene, caries, soft tissue diseases, socioeconomic factors, gender, the number of family members present, the presence of brothers or sisters, and cultural factors [17,18,19,20,21,22]. Some studies have shown that girls and younger children generally show higher levels of fear compared with boys and older children. Furthermore, children from families with lower socioeconomic status and education levels tend to have higher levels of dental anxiety [23]. According to published meta-analyses, the prevalence of dental fear and anxiety in children aged 2 to 6 years is 30%, while in preschool, primary school, and adolescent populations, the prevalence of dental anxiety is 36.5%, 25.8%, and 13.3%, respectively [23,24]. Another meta-analysis on the prevalence of dental fear and anxiety in children up to 19 years of age reports data ranging from 13.3% to 29.3%, depending on the method and test used to measure it [25].

Regarding the prevalence of dental anxiety and possible correlation factors, only a few relevant studies have been published in our country so far. A study on 1551 respondents in Croatia using an online questionnaire and online data collection showed that about 40% of respondents do not go to the dentist due to fear, and they also have a higher level of measured dental anxiety. Furthermore, about 55% of respondents have an elevated level of dental anxiety, which is above the median. Researchers concluded that dental anxiety depends on demographic, socioeconomic, and oral health factors. There is a clear connection between high dental anxiety scales and avoidance of going to the dentist due to acute pain [26].

The Modified Dental Anxiety Scale (MDAS) test has been frequently used in the analysis of dental anxiety in children, showing good reliability and criterion validity [27,28].

Respondents who had a previous bad experience associated with going to the dentist and who had moderate to severe dental anxiety achieved a statistically significantly higher score on the anxiety scale [6]. A previous study showed that active caries in children aged 6 years were statistically associated with maternal age, education, employment, and dental anxiety [29]. In contrast, another study showed that there was no proven correlation between the socioeconomic status of parents and dental anxiety in children [18]. Given that dental anxiety is a complex problem that depends on many factors that are not only of dental origin and that there are differences in dental anxiety levels between different people from different regions, additional research is needed to better understand this issue. We can state that dental anxiety also affects the quality of dental and general health, which could lead to public health issues.

The aim of this study is to analyze the prevalence of dental anxiety in a sample of Croatian patients who visit dental clinics, as well as the various factors associated with dental anxiety including the longitudinal aspect. The study hypothesis is that there is no difference in dental anxiety levels related to gender or socioeconomic status.

## 2. Materials and Methods

### 2.1. Research and Ethical Approval

This cross-sectional and longitudinal study was conducted within the School of Dental Medicine University of Zagreb, Croatia, and ethical approval was obtained under case number 003/01/25-05/04. This study was conducted from 2025 and 2026. Ethical approvals were obtained from private, public, and semi-private dental practices that participated in the study. All dental practices were familiar with the ethical protocol and the informed consent and patient and data protection procedures. Written information was provided for parents with all relevant facts about the study, and the same was explained to the children. Informed consent was signed by the parent or guardian/companion. The parents and the child could withdraw from the study at any time, without any consequences for further treatment as patients of the dental office.

### 2.2. Study Group

This study was conducted on a clinical sample of 150 children aged 12 to 18 years in the city of Zagreb and Zagreb County. The respondents were regular patients of dental practices in the area. The dental practices were considered public health, semi-private (contractual), and private practices based on their financing. Respondents were recruited from one private clinic, two public dental clinics and two contractual ones (semi-private), and the number of respondents was distributed relatively evenly. This research was conducted in three time intervals: T1 (before clinical examination), T2 (after clinical examination), and T3 (after a period of 3 months).

### 2.3. Inclusion Criteria

The inclusion criteria for children were the following:

○ Aged 12 to 18;
○ Reason for dental appointment: check-up or clinical examination;
○ Signed informed consent;
○ Native language of respondents and parents: Croatian;
○ Ability to understand the research and complete the questionnaire and test;
○ Patients at public health, semi-private (contractual), and private practices.

### 2.4. Exclusion Criteria

The exclusion criteria for children were the following:

○ Urgent dental procedures/pain.
○ Genetic malformations or some degree of intellectual disability.
○ Special needs.
○ Systemic diseases.
○ High degree of uncooperativeness.
○ Cannot speak and/or understand Croatian (including the parents).

### 2.5. Sample Analysis

#### Sample Size

The required minimum sample size was determined via power analysis (G*Power v.3.1.9,7, Heinrich Heine Universität Düsseldorf, Germany). Since patient acceptance for this study requires measurement at all three time points (T1, T2, T3), the required sample size must also consider the dropout of subjects. Therefore, we initially estimated (a priori) and rounded up the required sample size to 150 children for T1 and T2 (effect size d = 0.35; power: 90%; alpha significance level: 0.05; Wilcoxon Mann–Whitey test) measurements and 36 children for T3 measurement (effect size d = 0.51; power: 90%; alpha significance level: 0.05; Wilcoxon signed-rank test).

### 2.6. Data Collection and Questionnaire

Note that apart from the clinical examination/check-up and measurement of dental anxiety through the questionnaire, no other dental procedures were performed that could affect the level of dental anxiety (drilling, anesthesia, endodontic procedures, etc.). In case any dental pathology was observed during the examination, patients were referred for the next available appointment after they filled out the questionnaires, depending on the urgency of the procedure. The MDAS (Modified Dental Anxiety Scale) is a 5-item Likert-type scale [1,2,3,4,5] test that is summed to produce a total score ranging from 5 (no anxiety) to 25 points (maximum anxiety). Each item in the test is scored as follows: 1—not anxious; 2—slightly anxious; 3—moderately anxious; 4—highly anxious; 5—extremely highly anxious [30]. The Croatian version of the MDAS test has been verified and validated in previous studies [31,32].

The total MDAS cut-off scores that we used to categorize patients’ anxiety were as follows: 5–10, mild; 11–18, moderate; ≥19, severely anxious/possibly dental phobic. Therefore, a score of 19 points is the cut-off value, and higher scores are considered to correspond to high dental anxiety and possible dental phobia [33].

Children completed the questionnaire independently in the dental clinic, away from their parents, and parents had no influence on the completion of the general questionnaire, except in cases where there was some ambiguity about complex questions (for example, parents’ monthly income). The MDAS questionnaire was completed before the dental examination (T1), as was the general questionnaire. After the dental examination, which lasted approximately 10–15 min, the children completed the second MDAS questionnaire (the T2 questionnaire) independently in the dental office. After a period of three months, the children and parents were contacted to return for a regular dental examination, and then the T3 questionnaire was completed identically as before. A three-month period was chosen because it is the standard protocol in Croatia for dental check-ups in children, as well as in clinical practice; therefore, we consider it a sufficient time interval for re-examining anxiety.

The general questionnaire has 31 variables and collects information related to socioeconomic status (education, employment status, income, whether they live in a city or village), oral hygiene, dietary habits, and health satisfaction. Patient responses are scored and categorized into different categories such as low, medium, and high. Oral hygiene (OH) is categorized as low, medium, and high; oral status (OS) as poor, good, and excellent; cariogenic diet (CD) as low, medium, and high; and socioeconomic status (SES) as low, medium, and high. We categorized the responses from the questionnaire into categories so that we could further analyze them statistically. For example, the categories of oral hygiene (OH) are related to the scoring of answers from the questionnaire (frequency of brushing teeth, use of fluoride paste, use of dental floss, use of mouthwash, frequency of changing the brush, time of brushing teeth, etc.). Other groups (SES, CD, OS) were categorized in a similar manner. Additionally, data were collected at the practice where the examination was performed (private, semi-private, or public dental health; Zagreb or the surrounding area). Validity of questionnaire was verified on the basis of professional, clinical assessment experience of the chief examiner, a specialist in pedodontics (W.D.).

### 2.7. Multivariate Models

Questionnaire variables were recoded when necessary to form reasonably sized groups: sex (nominal, male/female), age (ordinal, 3 age groups), parents marital status (nominal, 3 groups), place of residence (nominal, urban/rural), health satisfaction (excluded due to low number of proper answers), oral heath satisfaction (nominal, yes/no), awareness of oral health measures (nominal, yes/no), financial impact on the use of dental services (nominal, yes/no), last visit to the dentist (ordinal, 3 groups), reason for last visit (nominal, regular/urgent), experiences with the dentist (ordinal, 2 groups) and type of dental service (nominal, 3 groups).

We recoded individual variables according to the low–medium–high scores, i.e., low was given 1 point, medium 2, and high 3, and these were later summed. Thus, numerical scores were obtained for SES, OH, OS, and CD. We used a similar questionnaire model with categorization of answers in a previous scientific study [34]. Given that such numerical scores can be interpreted as a total of low, medium, and high scores, in this study, we chose a pure numerical (higher is better) score in the regression analysis, i.e., we looked at the correlation of the score with the outcome variable (which is MDAS T1 in Model A, or the change in MDAS T2-T1 in Model B). For example, low family income or lack of oral hygiene beyond modern standards fell under a score of 1, while high family income and frequent and good oral hygiene received a higher score. A highly cariogenic diet was assigned 1 point, a moderately cariogenic diet 2 points, and a diet with low-cariogenic potential 3 points (higher scores indicating a more favorable profile). Subsequently, two questionnaire variables—frequency of sugar consumption and type of beverage consumed—were combined into a single CDn variable. Participants were then divided into three groups: high-cariogenic potential (2–3 points), moderate (4 points), and low (5–6 points). Finally, the correlation between the CDn variable and MDAS scores was calculated across various time intervals.

Compound variables were created by assigning points to questionnaire variables and adding them: SESn (education, employment, income)—scale ranging from 3 to 9; oral hygiene, OHn (frequency of brushing, technique for brushing, duration of brushing, floss, interdental brush, mouth rinse, fluoride toothpaste, changing toothbrush, toothbrush type)—scale ranging from 11 to 27; oral status, OSn (active caries, inflammation, breath, restored caries)—scale ranging from 4 to 12; cariogenic diet, CDn (sugar, beverages)—scale ranging from 2 to 6. Compound variables were scored based on the quality of the responses, so that the total responses were later categorized into categories such as SESn, OHn, OSn, and CDn to classify respondents into different levels. The principle was that poorer oral hygiene was assigned a lower number of points, while better oral hygiene was assigned a higher number of points. A similar principle applied to the other variables as well. For the CDn variable, a higher overall score indicated a better result in subsequent interpretations, given that a low-cariogenic diet is a desirable characteristic.

Outcome variables:

IMDAS—Initial MDAS score at T1.

FMDAS—Final MDAS score at T2.

RMDAS—Difference between final (T2) and initial direction (T2), a reduction in dental anxiety.

To model the outcome variables, two models were used.

**A. Model of association of independent variables with anxiety levels at T1**:

Dependent variable: MDAS before the clinical examination (T1).

Model: Sex, parents’ marital status, place of residence, awareness of oral health measures, financial impact on the use of dental services, time of last visit to the dentist, reason for last visit, type of dental service, experiences with the dentist, age of the respondent, SESn, OHn, OSn, CDn.

**B. Model of association of independent variables with the change in anxiety level between T1 and T2**: The difference between the MDAS score before and after the visit was used as the outcome variable, while the variables listed above were used as predictors. The MDAS difference score was calculated as T2-T1; thus, negative values represent reductions and positive values represent increases. Additionally, the IMDAS, which is the MDAS score at T1, was used as an additional continuous predictor.

Dependent variable: Change in MDAS score, T2-T1.

Model: (Intercept), sex of respondents, marital status of the parents, place of residence, awareness of oral health measures, financial impact on use of dental services, time of last visit to the dentist, reason for last visit, type of dental service, experiences with the dentist, age of respondents, SESn, OHn, OSn, CDn, MDAS before clinical examination (T1).

### 2.8. Statistical Analysis

Changes in dental anxiety across the different assessment time points were evaluated using the Friedman’s analysis of variance by ranks for related samples.

To investigate the influence of multiple independent variables on dental anxiety, two generalized linear models (GLMs) were constructed. **Model A** was used to identify factors independently associated with the MDAS score at T1, thereby examining factors related to the level of dental fear. **Model B** was used to identify factors associated with the change in MDAS score between T1 and T2 (T2–T1), thereby examining factors related to changes in dental fear.

The internal consistency of the Croatian version of the MDAS was assessed using Cronbach’s alpha. Test–retest reliability was evaluated using Spearman correlation coefficient for individual questionnaire variables and summary scales.

Microsoft Office Excel for Windows (Microsoft Corporation, Redmond, WA, USA) was used for data entry and graph creation. The data were statistically analyzed using SPSS 20.0 for Windows (SPSS Inc., Chicago, IL, USA) with a statistical significance level α of 0.05, i.e., a confidence level of 95%.

## 3. Results

### 3.1. Test Analysis

Cronbach’s alpha for the MDAS was 0.816, which is sufficient for scientific work. The correlation between the results at T1 and T2 was statistically significant (r = 0.522, Spearman’s correlation coefficient, *p* < 0.001). Such a correlation indicates that 27% of the variance of the MDAS variable is stable, i.e., a permanent trait, while the rest of the variance is influenced by other circumstances.

### 3.2. Demographics

There were 84 female children (56%) and 66 male children (44%). Regarding the age groups, there were 82 (54.6%) children in the 12–13 age group, 27 (18%) in the 14–15 age group, and 41 in the 16–18 (27.4%) age group. In total, 44% of the children were from public dental practice, 24.6% from private dental practice, and 31.3% from contracted (semi-private) practice.

No gender-related statistical difference in dental anxiety was confirmed at T1 and T2; therefore, we can accept the original hypothesis. Between children living in the city and rural areas, there was no statistical difference in dental anxiety, although anxiety was slightly higher in children from rural areas (9.84 vs. 10.36).

### 3.3. Results of MDAS Test at Different Time Intervals/Longitudinal Component

**Figure 1:**
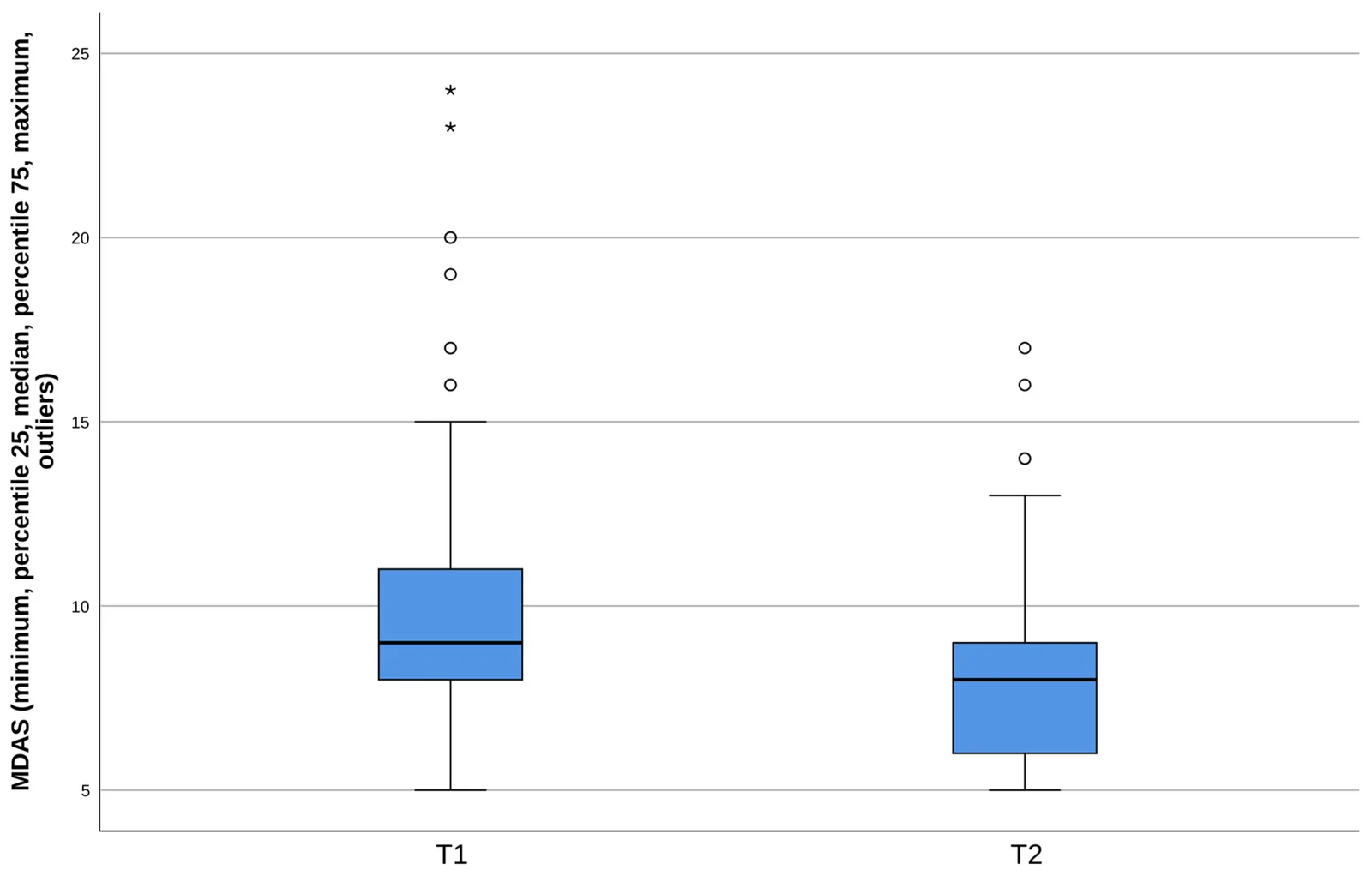
Differences in MDAS at T1 and T2. o—outliers (exceeding 1.5 IQR), *—extreme outliers (exceeding 3 IQR).

There are statistically significant differences between the MDAS test results before and after the procedure (T1 vs. T2) in the way that dental anxiety decreased in most children (Friedman’s ANOVA, *p* < 0.001). Figure 1 shows the differences at different time periods.

Table 1 shows that the average value of the MDAS test was 9.79 for T1, and 8.03 for T2, which indicates mild anxiety. Before the clinical examination (T1), children mostly had mild dental anxiety (55.3%), followed by moderate (41.3%) and severe dental anxiety (3.4%). After the clinical examination (T2), children mostly had mild dental anxiety (77.2%), followed by moderate anxiety (22.8%) and no severe anxiety.

Of the initial N = 150 children, N = 61 responded at the T3 period (after 3 months).

The required minimum sample size was determined via power analysis (G*Power, v.3.1.9,7, Heinrich Heine Universität Düsseldorf, Germany). Since patient acceptance for this study requires measurement at all three time points (T1, T2, T3), the required sample size must also consider the dropout of subjects. Therefore, we initially estimated (a priori) and rounded up the required sample size to 150 children for T1 and T2 (effect size d = 0.35; power: 90%; alpha significance level: 0.05) measurements and 36 children for T3 measurement (effect size d = 0.51; power: 90%; alpha significance level: 0.05: Wilcoxon signed-rank test).

The differences in T1, T2 and T3 were analyzed on the subsample (n = 61) of patients who had all the data, so that they can be analyzed using related sample analysis, comparing the T3 respondents to themselves at earlier time points. The relationship between dental fear and the reduction in fear after the dental visit compared to before the visit was analyzed on a larger sample of n = 150.

**Figure 2:**
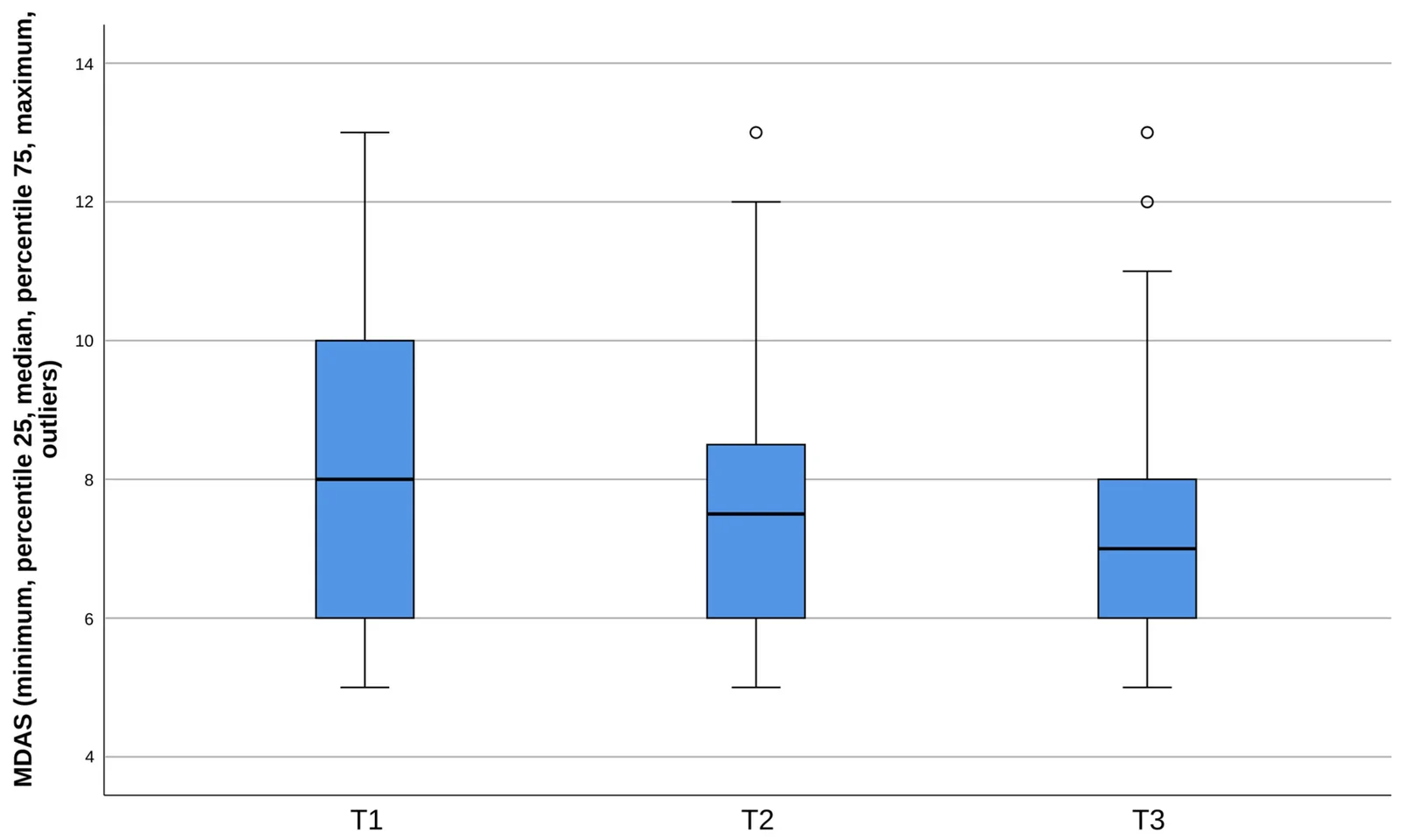
Differences in MDAS at T1, T2, and T3. o—outliers (exceeding 1.5 IQR).

There are statistically significant differences between individual time points T1, T2, and T3 (*p* < 0.001), and these differences are significant between T1 and T2 (*p* = 0.045) and between T1 and T3 (*p* = 0.012). Between T2 and T3, the differences were not statistically significant (*p* = 0.616). The results are shown in Table 2 and Figure 2.

**Figure 3:**
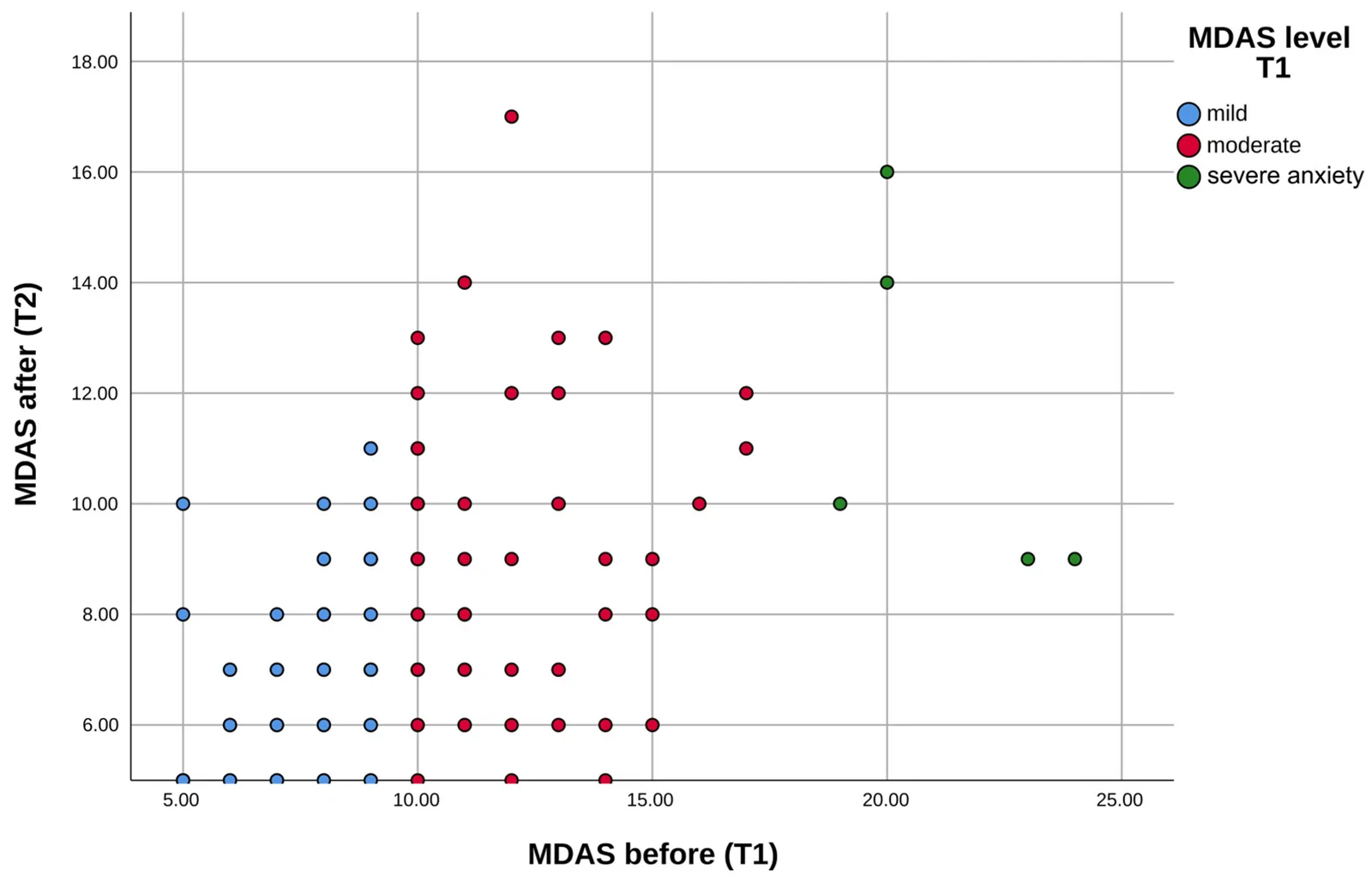
Grouped scatter plot of MDAS scores after treatment (T2) vs. before treatment (T1) with anxiety level.

Children with mild dental anxiety before the clinical examination (T1) have mild dental anxiety after the clinical examination (T2) in 92% of the cases. Children who have moderate dental anxiety before the clinical examination (T1) have mild or moderate dental anxiety in 61% or 39% of the cases, respectively, after the clinical examination. Severe anxiety in children before the clinical examination (T1) decreases to mild or moderate levels afterwards (T2) (Figure 3).

### 3.4. Multivariate Model Analysis of Model A

Due to insufficient data and categories that had to be excluded, a total of N = 108 respondents were included in Model A. Specifically, many cases involved omissions during questionnaire completion, data entry errors, multiple responses, and similar writing errors. The total MDAS scores before and after deviate statistically significantly from the normal distribution (Kolmogorov–Smirnov test, *p* < 0.001, Friedman’s ANOVA by ranks).

The model itself is statistically significant, with *p* = 0.021 in total, and there are significant effects within the model: financial influence on the use of dental services (*p* = 0.014), type of dental service (*p* = 0.008), and experience with the dentist (*p* = 0.004). A negative correlation with the MDAS score before the clinical examination was found in respondents who reported “financial impact” on the use of dental services = “yes” and type of dental service = “public”, and who have “positive experience with the dentist”. Other variables do not show a statistically significant influence on the model (Table 3). We can conclude that anxiety level before treatment (T1) is influenced by financial impact (54% of the responses were “yes”), the type of dental service (39.8% of the answers were “public dental service”), and previous experiences with the dentist (75.9% of the responses were “positive experiences”).

### 3.5. Multivariate Model Analysis of Model B

The model is statistically significant, with *p* < 0.001 in total, and the following effects are significant: MDAS score at T1 (*p* < 0.001), financial impact on the use of dental services (*p* < 0.006), experiences with the dentist (*p* < 0.046) and CDn (*p* < 0.041). The initial MDAS and CDn scores have a negative correlation with the change in the MDAS score, i.e., greater fear before the clinical examination (T1) is associated with a greater reduction in fear after the clinical examination (T2). Children who had a better score for cariogenic diet (CDn), indicating a low level of cariogenic food in their daily consumption, are associated with a greater reduction in fear after the clinical examination (T2).

The results are shown in Table 4.

### 3.6. Analysis of Responses from Questionnaires Related to Lifestyle Habits

Regarding the details of oral hygiene (OH), 56% of the children brush their teeth once a day or less, 62% do not floss, 74% do not use an interdental brush, 66% do not use mouthwash, and 28% of the children use the same toothbrush for more than three months. We can conclude that children have poor oral hygiene habits by modern standards, and there is a potential for an increase in the incidence of caries given such oral hygiene results.

Regarding oral status (OS), 49.3% of the children have more than one active carious lesion, 44% have gum inflammation, and 38.6% have bad breath, which indicate that oral status is not satisfactory among the children of this age.

Regarding cariogenic diet (CD), 44.6% of children frequently consume sweets and sugar, 36.6% drink sweetened beverages, and 22.6% smoke (electronic vape or tobacco). These results also indicate a possible high risk of developing caries.

Regarding socioeconomic status (SES), 40% of respondents come from families where one or both parents have a college degree or a master’s/doctorate degree, 93% of them are employed, and 82% live in the city, but only 15% have above-average incomes, i.e., a net salary higher than 2500 euros in accordance with their higher education.

## 4. Discussion

The MDAS test has been used for more than thirty years to analyze dental anxiety. Nonetheless, there are some limitations in the interpretation of the test results, as well as in the inconsistency of the research, especially in the cut-off values for dental anxiety. Ideally, researchers should provide adequate descriptions of the research tests used and properly cite references so that the methodology can be standardized and compared with other works.

The total MDAS score should be reported to allow comparison with previous research and to provide an indication of respondents having a particularly high score (above 19 out of a maximum of 25). Subscale statistics can be tabulated to provide additional information on two potentially important and theoretically defined features of dental anxiety: anticipatory and treatment-related features [35]. The report of all published MDAS studies for the period 2014–2023 showed that there were large variations in the methodology itself, and they significantly deviated from the original version of the MDAS test. Many differences were discovered from the original version, and the most significant one was using a threshold value that differed from the original, using more than one limit value, modifying the response format or descriptors, and modifying question items, especially the inclusion of additional questions. All these actions would create confusion if researchers and clinicians try to compare data across different studies. Therefore, it is recommended for researchers to apply MDAS in its original format without modifications, as performed in this study [28].

Global research on dental anxiety in children shows us that it is a common phenomenon in children, it varies between countries and different cultures, and it depends on many factors. According to a meta-analysis of published papers from 2000 to 2023 on DFA (dental fear and anxiety), 30% of preschool children have this problem. The authors concluded that children who had never been to the dentist had a high DFA, suggesting that regular dental visits may reduce the risk. Children with dental caries were also at risk. The research did not show a significant statistical difference in DFA between boys and girls [24]. Given that these were children of younger age (2–6 years), different tests and cut-offs were used to measure dental anxiety. The studies demonstrated different heterogeneity, and thus, the results must be taken with caution if compared with those of our study. In our study, according to the results of the MDAS test, 3.3% of the children have a high level of dental anxiety, while 41.3% of the children have moderate anxiety.

Another study analyzed the published scientific literature to quantify the prevalence and mean score of dental fear/anxiety (DFA) in children/adolescents and its variations according to several variables. The prevalence of DFA measured with MDAS tests among the studies varied from 13.3% to 29.3%. The mean score of DFA ranged from 18.1 to 20.81. In the studies employing the CFSS-DS scale, younger and female children had higher DFA values. Northern European children and adolescents had a lower prevalence and lower levels of DFA compared with their peers in other geographical areas. If we look at the MDAS test results of four studies, DFA prevalence in children aged 7 to 16 was 12.2%, and the mean score was 12.5, which is somewhat comparable with our results. We obtained a mean score of 9.79 in children aged 12–18 years, at the T1 interval. In general, at least one child out of ten had a level of DFA that hindered their dental treatment [25]. Also, the multifactorial etiology of children’s dental fear and anxiety identifies the influence of many different risk factors in its development [36].

The association between dental anxiety and dental caries in primary teeth and younger children was established in a systematic review and meta-analysis. A possible explanation is that younger children are less socially developed and dental fear is more obvious in primary teeth than permanent ones, which come in at older ages [37]. Dental fear is associated with a lack of communication between the dentist and the child in the first year, which leads to dental caries and further treatment avoidance [38]. In our study, 49.3% of the children have one or more active caries lesions. The questionnaire results show that there were other indicators of poor outcomes related to OH, CD, and OS. It is possible that poor oral health, diet, and poor oral hygiene indicators—by modern standards—may influence dental anxiety. Other authors have confirmed an increase in dental anxiety in children aged 7 to 9 years, and the risk factors were new carious lesions (four-times-higher odds ratio), pain (four-times-higher odds ratio), and extractions (five-times-higher odds ratio), which mean that dental treatment should focus on the prevention of caries and traumatic experiences [39]. Note that unpleasant experiences in the dental office during childhood remain a lasting and significant factor (21.7 times odds ratio) in avoiding and canceling dental treatments in adulthood [8]. Research on 125 children aged 9 to 12 years showed that dental anxiety was lower in children who underwent orthodontic treatment than in those who underwent tooth extraction or received local anesthesia. The results indicate construct validity for the MDAS according to the treatment received by children who had a history of orthodontic procedures and thus, lower dental anxiety, primarily due to the frequency of orthodontic appointments and a greater number of interventions, which acted as desensitization [29]. Furthermore, a study on 203 children aged 5 to 18 years showed elevated scores in all anxiety-related variables when comparing first dental visit vs. previous visits, as well as differences in anxiety based on age (younger children presented higher levels), gender differences (female children are more anxious), and cultural differences between nations [40]. Therefore, the importance of regular dental visits may be one possible factor for the prevalence of dental anxiety, as it is well known that regular dental check-ups are crucial for maintaining good oral health.

Regarding the analysis of dental anxiety before and after treatment, there is an insufficient number of published papers on this issue. Only one scientific paper from AlAzmah et al. was similar to our study. In their study on 200 children aged 6 to 9, they measured dental anxiety using the MDAS and STAI tests before and after a dental procedure. Their results showed that the mean MDAS value was 14.54 and 9.4 before and after the procedure, respectively, which is statistically significant. Among the children, 38% experienced “extreme” anxiety; 13% experienced “mild” anxiety; and 49% experienced “no anxiety” [41]. A possible explanation for the high level of dental anxiety is the first initial dental appointment, and that they did not measure any association with other causes such as socioeconomic factors. Furthermore, it is important to note that dental anxiety decreased after the procedure. This was also demonstrated in our study, where the mean MDAS value was slightly lower than in the study by AlAzmah et al. The mean MDAS value was 8.17 before the clinical examination, which decreased to 7.61 after the clinical examination, a statistically significant decrease. Furthermore, after 3 months, the mean MDAS value was 7.54. We can conclude that dental anxiety does decrease over time and that regular and frequent appointments are important. A review of the scientific literature revealed no similar studies incorporating a longitudinal component in the assessment of dental anxiety in children using the MDAS questionnaire; consequently, this study represents a novel contribution and a model for future research. We recommend further studies employing various time intervals—involving both children and adults—to clarify the impact of the passage of time on the level of dental anxiety.

A high DMFT index in children was 2.64 times associated with dental fear, as were gingivitis and poor oral hygiene, the association rates of which were as high as 4.95 and 4.93 times, respectively [21]. Given that the prevalence of caries in children in Croatia is also high, we can agree with these results and confirm the association of poor oral status and hygiene with dental anxiety in children.

A study on 400 children aged 6–12 years in India showed that 23% of children had mild anxiety, and as many as 61.5% had severe anxiety, but these levels decreased with age. The average anxiety in children aged 12 was 15.38, and in children aged 13, it was 14.46, which was higher than our result of 10.24. A possible explanation is cultural differences and the fact that there were more female children in the study, who have been shown to have higher levels of dental anxiety [42].

Regarding the type of dental practice, the results from our study show a significant difference in levels of dental anxiety between public dental service and other types of dental service. Nonetheless, there are possible limitations affecting the final results mentioned here, as the largest number of respondents was from public dental clinics (44%), which may affect the interpretation of the results. In contrast, a study on 280 children aged 6 to 12 years showed that dental fear and anxiety were significantly higher in the private dental setting compared with the public dental setting [43]. We can agree that a possible explanation is the fact that families and children who visited a public dental hospital and public dental service have a low socioeconomic status but exhibit a lower prevalence of dental fear because they are less educated and more aware of their oral health [44]. Patients who belong to a higher socioeconomic status are generally more afraid at the dental clinic [45]. Further studies are needed to establish a strong correlation between low socioeconomic status and low dental fear. Given that a lower level of dental anxiety is associated with regular dental examinations/check-ups and the absence of negative dental experiences (pain, emergencies), these conclusions should be considered by clinicians and implemented in preventive dental programs for children. As we additionally used the multivariable explanatory model for analysis, the results show that there is statistical significance in the influence of finances on the choice of dental services, the type of dental service provider in relation to financing, and previous positive experiences with the dentist. However, no statistically significant difference was found between dental anxiety and gender or socioeconomic status. Model B of the multivariable analysis, as shown before, demonstrated a decrease in dental anxiety in the T2 interval related to compound variables such as cariogenic diet (CDn), experiences with the dentist, and financial impact on the use of dental services. In individuals who had a higher dental anxiety score before the clinical examination (T1), a greater decrease in dental anxiety score was observed after the clinical examination (T2). This may be potentially interpreted as visits to the dentist may have influence in reducing the level of dental anxiety, if the children do not experience painful or traumatic dental procedures. Although a high CDn score indicates a good diet, low in sugar and cariogenic foods, it is possible that individuals in this group experience slightly higher anxiety reduction to the awareness of their good dietary habits and possible good oral health; however, as we have demonstrated, the level of dental anxiety decreases following a dental check-up. Less-cariogenic diet may be associated with greater reduction in fear after the procedure (T2). Also, it is possible that their fear is more situational. Children with generally higher anxiety levels may consume more sweets and unhealthy foods, what they can be aware of, resulting in a smaller reduction in dental anxiety over time. We can conclude that positive previous experiences with the dentist, as well as healthy oral hygiene and dietary habits, may have an impact on the level of dental anxiety.

In our study, children were only accompanied by their parents due to the legal obligations for minor patients, and the parents were not present during the completion of the questionnaire or the dental examination/check-up. This may mean that there was no parental influence during the analysis of dental anxiety. The presence of parents affects the child’s behavior and can enhance behavior in specific subgroups but lacks universal benefits [46]. Studies have confirmed the influence of parents on completing dental anxiety tests, including telephone and at-home questionnaires, which can lead to inaccurate results [16,47,48]. Meta-research has confirmed that the presence of parents does not affect the behavior of children under 12 years of age, nor their fears or anxiety during dental treatment; however, the evidence from the analyzed studies has only provided low levels of certainty [49]. The strength of our study is that a cut-off point of 19 for high dental anxiety was used, as originally proposed for the MDAS test. Furthermore, the children were separated from their parents during the examination to fill out the questionnaire so that they would not be influenced. The sample size was adequate, and this is the first study to analyze DFA in children at three different intervals, enabling future longitudinal studies to be conducted on dental anxiety in children. Even if a study has a large-enough number of events per independent variable, the estimates of the association between a risk factor and an outcome may still be inaccurate if the risk factor is rare. In addition, a sufficient sample size does not guarantee that results from a model can be reproduced with new data [50].

It is important to note several limitations of our study. First, it is a known limitation of cross-sectional studies that causal interference cannot be determined and that confounding variables may influence the results. Here, the MDAS should be considered the first screening tool for trait-focused anxiety assessment, while situational tools such as the STAI-State/VAS-Anxiety should be used to assess periprocedural (state) anxiety as part of the clinical course. Although the advantages of using causal models might not yet be fully appreciated in dental research, they should be considered as essential tools for clarifying complex inter-relationships between variables [51].

Second, the use of a self-report questionnaire is a potential source of bias. Third, the children’s temperament, the parental style, and the relationship between parents and children were not analyzed. Fourth, given the differences in the number of patients in different groups at different clinics, there are possible limitations in the final results, given that most of the children attended public clinics. Additional limitations include participant dropout by T3 and participants from Zagreb and Zagreb County not being representative of the entire population given that we conducted the study on a clinical sample of patients. It is important to note that the results of the subjects in the longitudinal T1-T3 period refer only to the number of subjects of 61, out of the initial 150. Therefore, there are possible methodological limitations of the study due to the loss of patients as well as the impossibility of them not being assessed or excluded.

In multivariable explanatory model analysis, reliability means that a different dataset would likely yield a model with the same variables and similar coefficients. Given the slightly smaller number of respondents in this analysis in our study, we must also interpret the results with caution.

## 5. Conclusions

The examined children exhibited mild to moderate anxiety according to the MDAS test, which tended to decrease after a dental clinical examination. There is no statistically significant difference between dental anxiety and gender or socioeconomic status. Financial impact on the use of dental services, type of dental service, experiences with the dentist and cariogenic diet may be associated with dental anxiety at different time periods. Differences in dental anxiety levels were found between public dental services and other types of dental services. More frequent dental check-ups within a three-month period could be presented as a hypothesis for future studies when analyzing the reduction in dental anxiety in children.

## Figures and Tables

**Table 1:** The average values and differences in MDAS values at time intervals T1 and T2 (N = 150).

	MDAS T1	MDAS T2
Mean	9.79 *	8.03 *
Standard Deviation	3.40	2.43
Median	9.00	8.00
Minimum	5.00	5.00
Maximum	24.00	17.00
25th Percentile	8.00	6.00
75th Percentile	11.00	9.00
Count	150	150

* Statistical significance, *p* < 0.001.

**Table 2:** The average values and differences in MDAS values at time intervals T1, T2, and T3 (N = 61).

	MDAS T1	MDAS T2	MDAS T3
Mean	8.17 *^§†^	7.61 *^†^	7.54 *^§†^
Standard Deviation	2.32	1.98	2.05
Median	8.00	8.00	7.00
25th Percentile	6.00	6.00	6.00
75th Percentile	10.00	9.00	8.00
Count	61	61	61

* Statistical significance, *p* = 0.045. ^§^ Statistical significance, *p* = 0.012. ^†^ Statistical significance, *p* < 0.001.

**Table 3:** Multivariate model analysis results for Model A.

	B	Std. Error	95% Wald Confidence Interval		Hypothesis Test		
			Lower	Upper	Wald Chi-Square	df	Sig
Intercept	16.806	2.5442	11.819	21.793	43.634	1	0.000
Sex = 1.00	−0.215	0.6566	−1.502	1.071	0.108	1	0.743
Sex = 2.00	0 ^a^						
Parents’ marital status = 1.00	−0.124	0.8407	−1.772	1.523	0.022	1	0.882
Parents’ marital status = 2.00	−0.648	1.4904	−3.569	2.273	0.189	1	0.664
Parents’ marital status = 3.00	0 ^a^						
Place of residence = 1.00	0.836	0.8252	−0.781	2.453	1.027	1	0.311
Place of residence = 2.00	0 ^a^						
Awareness of oral health measures = 1.00	−0.917	0.7962	−2.477	0.644	1.326	1	0.250
Awareness of oral health measures = 2.00	0 ^a^						
Financial impact on the use of dental services = 1.00	−1.675	0.6815	−3.011	−0.339	6.041	1	0.014 *
Financial impact on the use of dental services = 2.00	0 ^a^						
Last visit to the dentist = 1.0	−1.768	0.8807	−3.494	−0.042	4.031	1	0.045
Last visit to the dentist = 2.0	−1.460	1.0175	−3.454	0.534	2.059	1	0.151
Last visit to the dentist = 3.0	0 ^a^						
Reason for last visit = 1.00	0.314	0.8134	−1.280	1.908	0.149	1	0.700
Reason for last visit = 2.00	0 ^a^						
Type of dental service = 1.00	−1.953	0.7397	−3.403	−0.503	6.968	1	0.008 *
Type of dental service = 2.00	−0.525	0.8679	−2.226	1.176	0.366	1	0.545
Type of dental service = 3.00	0 ^a^						
Experiences with the dentist = 1.00	−2.577	0.8942	−4.330	−0.824	8.305	1	0.004 *
Experiences with the dentist = 2.00	0 ^a^						
Age	−0.295	0.3665	−1.013	0.424	0.647	1	0.421
SESn	−0.422	0.2561	−0.924	0.080	2.718	1	0.099
OHn	−0.070	0.1169	−0.299	0.159	0.358	1	0.550
OSn	0.298	0.1720	−0.039	0.635	3.001	1	0.083
CDn	0.131	0.3065	−0.470	0.732	0.182	1	0.670
Scale	8.569 ^b^	1.1660	6.563	11.188			

a. Set to zero because this parameter is redundant. b. Maximum likelihood estimate. * Statistical significance.

**Table 4:** Multivariate model analysis results for Model B.

	B	Std. Error	95% Wald Confidence Interval		Hypothesis Test		
			Lower	Upper	Wald Chi-Square	df	Sig.
Intercept	−3.813	2.3218	−8.364	0.738	2.697	1	0.101
Sex = 1.00	0.297	0.5992	−0.878	1.471	0.245	1	0.621
Sex = 2.00	0 ^a^						
Parents’ marital status = 1.00	0.747	0.7672	−0.757	2.251	0.948	1	0.330
Parents’ marital status = 2.00	0.198	1.3601	−2.468	2.863	0.021	1	0.884
Parents’ marital status = 3.00	0 ^a^						
Place of residence = 1.00	0.400	0.7531	−1.076	1.876	0.282	1	0.595
Place of residence = 2.00	0 ^a^						
Awareness of oral health measures = 1.00	1.133	0.7266	−0.291	2.557	2.433	1	0.119
Awareness of oral health measures = 2.00	0 ^a^						
Financial impact on the use of dental services = 1.00	1.719	0.6219	0.500	2.938	7.638	1	0.006 *
Financial impact on the use of dental services = 2.00	0 ^a^						
Last visit to the dentist = 1.0	−0.211	0.8037	−1.786	1.365	0.069	1	0.793
Last visit to the dentist = 2.0	−1.026	0.9285	−2.846	0.794	1.221	1	0.269
Last visit to the dentist = 3.0	0 ^a^						
Reason for last visit = 1.00	−0.720	0.7423	−2.175	0.735	0.942	1	0.332
Reason for last visit = 2.00	0 ^a^						
Type of dental service = 1.00	0.913	0.6751	−0.410	2.236	1.829	1	0.176
Type of dental service = 2.00	0.875	0.7920	−0.677	2.427	1.221	1	0.269
Type of dental service = 3.00	0 ^a^						
Experiences with the dentist = 1.00	1.628	0.8161	0.028	3.227	3.979	1	0.046 *
Experiences with the dentist = 2.00	0 ^a^						
Age	−0.171	0.3345	−0.827	0.484	0.262	1	0.609
SESn	0.180	0.2337	−0.278	0.638	0.594	1	0.441
OHn	0.045	0.1067	−0.164	0.254	0.178	1	0.673
OSn	−0.160	0.1570	−0.467	0.148	1.033	1	0.309
CDn	−0.572	0.2797	−1.120	−0.024	4.183	1	0.041 *
Scale	7.136 ^b^	0.9711	5.466	9.317			

a. Set to zero because this parameter is redundant. b. Maximum likelihood estimate. * Statistical significance.

## Data Availability

The data presented in this study are available on request from the corresponding authors, excluding private data from patients or data that conflict with ethical propositions or patient data protection.

## Abbreviations

The following abbreviations are used in this manuscript:

SD	Standard deviation
T1	Time interval before clinical examination
T2	Time interval after clinical examination
T3	Time interval after 3 months
OH	Oral hygiene
OS	Oral status
SES	Socioeconomic status
CD	Cariogenic diet
OHn	Oral hygiene in Model A statistical analysis
OSn	Oral status in Model A statistical analysis
SESn	Socioeconomic status in Model A statistical analysis
CDn	Cariogenic diet in Model A statistical analysis
DFA	Dental fear and anxiety
MDAS	Modified Dental Anxiety Scale test
DMFT	Decayed Missed Filled Teeth index

## References

[1] Armfield J.M., Heaton L.J. (2013). Management of fear and anxiety in the dental clinic: A review. Aust. Dent. J..

[2] Bürklein S., Brodowski C., Fliegel E., Jöhren H.P., Enkling N. (2021). Recognizing and differentiating dental anxiety from dental phobia in adults: A systematic review based on the German guideline “Dental anxiety in adults”. Quintessence Int..

[3] Klingberg G., Brober A.G. (2007). Dental fear⁄anxiety and dental behaviour management problems in children and adolescents: A review of prevalence and concomitant psychological factors. Int. J. Paediatr. Dent..

[4] Klaassen M.A., Veerkamp J.S., Aartman I.H., Hoogstraten J. (2002). Stressful situations for toddlers: Indications for dental anxiety?. ASDC J. Dent. Child.

[5] Yamada M.K., Tanabe Y., Sano T., Noda T. (2002). Cooperation during dental treatment: The Children’s Fear Survey Schedule in Japanese children. Int. J. Paediatr. Dent..

[6] Surina K., Ruždijić J., Kuzinovska A., Stevanović A., Rončević-Gržeta I. (2021). Strah od odlaska stomatologu. Soc. Psihijat.

[7] Armfield J.M., Stewart J.F., Spencer A.J. (2007). The vicious cycle of dental fear: Exploring the interplay between oral health, service utilization and dental fear. BMC Oral Health.

[8] Åstrøm A.N., Agdal M.L., Sulo G. (2024). Exploring avoidance of dental care due to dental fear and economic burden -A cross-sectional study in a national sample of younger adults in Norway. Int. J. Dent. Hyg..

[9] Liinavuori A., Tolvanen M., Pohjola V., Lahti S. (2016). Changes in dental fear among Finnish adults: A national survey. Community Dent. Oral Epidemiol..

[10] Dou L., Vanschaayk M.M., Zhang Y., Fu X., Ji P., Yang D. (2018). The prevalence of dental anxiety and its association with pain and other variables among adult patients with irreversible pulpitis. BMC Oral Health.

[11] Sukumaran I., Taylor S., Thomson W.M. (2020). The prevalence and impact of dental anxiety among adult New Zealanders. Int. Dent. J..

[12] Sivaramakrishnan G., Makki H., AlDallal S., Alaswad Z., Sultan E., Ahmed S., AlBanna H., Alsobaiei M., AlSalihi L. (2022). The variables associated with dental anxiety and their management in primary care dental clinics in Bahrain: A cross-sectional study. BMC Oral Health.

[13] Humphris G., Spyt J., Herbison A.G., Kelsey T.W. (2016). Adult dental anxiety: Recent assessment approaches and psychological management in a dental practice setting. Dent. Update.

[14] Leko J., Škrinjarić T., Goršeta K. (2020). Reliability and validity of scales for assessing child dental fear and anxiety. Acta Stomatol. Croat..

[15] Lara A., Crego A., Romero-Maroto M. (2012). Emotional contagion of dental fear to children: The fathers’ mediating role in parental transfer of fear. Int. J. Paediatr. Dent..

[16] Jervøe-Storm P.M., Peters L.P., Bekes K., Fricke M., Jepsen S. (2023). Evaluation of Children’s Anxiety Level in Relation to a Dental Visit/Treatment and Their Parents’ Dental Fear. J. Clin. Med..

[17] Mendoza-Mendoza A., Perea M.B., Yañez-Vico R.M., Iglesias-Linares A. (2015). Dental fear in children: The role of previous negative dental experiences. Clin. Oral Investig..

[18] Petrović D., Cicvarić O., Šimunović-Erpušina M., Ivančić Jokić N., Bakarčić D., Bučević Sojčić P., Jurić H. (2024). The Role of Family Factors in the Development of Dental Anxiety in Children. Medicina.

[19] Grisolia B.M., Dos Santos A.P.P., Dhyppolito I.M., Buchanan H., Hill K., Oliveira B.H. (2021). Prevalence of dental anxiety in children and adolescents globally: A systematic review with meta-analyses. Int. J. Paediatr. Dent..

[20] Alshoraim M.A., El-Housseiny A.A., Farsi N.M., Felemban O.M., Alamoudi N.M., Alandejani A.A. (2018). Effects of child characteristics and dental history on dental fear: Cross-sectional study. BMC Oral Health.

[21] Eric J., Davidovic B., Mladenovic R., Milosavljevic M., Miljevic I.D., Bjelovic L., Jankovic S., Dolic O., Davidovic B. (2025). Prevalence of Dental Fear and Its Association with Oral Health Status Among School Children in Bosnia and Herzegovina: A Cross-Sectional Study. Medicina.

[22] Gao X. (2018). Children’s dental fear and anxiety: Exploring family related factors. BMC Oral Health.

[23] Dogan M.C., Seydaoglu G., Uguz S., Inanc B.Y. (2006). The effect of age, gender and socio-economic factors on perceived dental anxiety determined by a modified scale in children. Oral Health Prev. Dent..

[24] Sun I.G., Chu C.H., Lo E.C.M., Duangthip D. (2024). Global prevalence of early childhood dental fear and anxiety: A systematic review and meta-analysis. J. Dent..

[25] Cianetti S., Lombardo G., Lupatelli E., Pagano S., Abraha I., Montedori A., Caruso S., Gatto R., De Giorgio S., Salvato R. (2017). Dental fear/anxiety among children and adolescents. A systematic review. Eur. J. Paediatr. Dent..

[26] Peric R., Tadin A. (2024). Associations between Dental Anxiety Levels, Self-Reported Oral Health, Previous Unpleasant Dental Experiences, and Behavioural Reactions in Dental Settings: An Adult E-Survey. Medicina.

[27] Lahti S., Kajita M., Pohjola V., Suominen A. (2025). Reliability and Validity of the Modified Dental Anxiety Scale Among Children Aged 9 to 12 Years. Dent. J..

[28] Yeung A.W.K. (2024). Exploring the Variations in the Use of Modified Dental Anxiety Scale (MDAS) in Literature: A Survey of Studies Published from 2014 to 2023. Clin. Exp. Dent. Res..

[29] Gavic L., Tadin A., Matkovic A., Gorseta K., Sidhu S.K. (2022). The association of parental dental anxiety and knowledge of caries preventive measures with psychological profiles and children’s oral health. Eur. J. Paediatr. Dent..

[30] Humphris G.M., Morrison T., Lindsay S.J. (1995). The Modified Dental Anxiety Scale: Validation and United Kingdom norms. Community Dent. Health.

[31] Čabraja L.K., Lapaš-Barišić Z.M., Dukić W. (2026). Prevalence of dental anxiety and correlation with other factors in a sample of adult Patients. Acta Stomatol Croat..

[32] Čabraja L.K. (2025). Evaluation of the MDAS/MDAS-DEP tests for measuring dental anxiety: A pilot study. Acta Stomatol Croat..

[33] UK Government Clinical Standards for Dental Anxiety Management. National Health Service (NHS) 2023. https://www.england.nhs.uk/long-read/clinical-guide-for-dental-anxiety-management/.

[34] Lešić S. (2019). Oral Health Factors of School Children in Urban and Rural Areas. Ph.D. Thesis.

[35] Humphris G.M., Newton J.T. (2025). Is the Modified Dental Anxiety Scale (MDAS) a Single or Two Construct Measure? A Theoretical and Pragmatic Perspective. Dent. J..

[36] Shindova M.P., Belcheva A.B. (2021). Dental fear and anxiety in children: A review of the environmental factors. Folia Med..

[37] Amrollahi N., Shahshahan S.A., Nilchian F., Tarrahi M.J. (2024). Dental Fear and Caries in 6- to 12-Year-Old Children: A Systematic Review and Meta-analysis. Chin. J. Dent. Res..

[38] Inglehart M.R. (2019). Motivational Communication in Dental Practices: Prevention and Management of Caries over the Life Course. Dent. Clin. N. Am..

[39] Dahlander A., Soares F., Grindefjord M., Dahllöf G. (2019). Factors Associated with Dental Fear and Anxiety in Children Aged 7 to 9 Years. Dent. J..

[40] Borrell Garcia C., Boo Gordillo P., Garcia Miralles E., Aura Tormos J.I., Guinot Barona C., Marqués Martínez L. (2026). Psychological Factors in Paediatric Dentistry: A Cross-Sectional Study. Eur. J. Paediatr. Dent..

[41] AlAzmah A., Sharanesha R.B., Abushanan A., Khojah A.B., Dhaafi A.A., Almakenzi A.A., Alqarni A.S., Alagla M., Al Ghwainem A., Alghamdi S. (2024). Comparison of Parental and Children’s Dental Anxiety Levels Using the Modified Dental Anxiety Scale and Modified Short State-Trait Anxiety Inventory (EMOJI) Scale. Children.

[42] Kumar V., Goud E.V.S.S., Turagam N., Mudrakola D.P., Ealla K.K.R., Bhoopathi P.H. (2019). Prevalence of Dental Anxiety Level in 6- to 12-Year- Old South Indian Children. J. Pharm. Bioallied Sci..

[43] Mobin T., Khan T.Z., Mobin A., Tahir M.R., Imran Q., Gardezi S.A.M., Waqar R., Hanif M., Mohamed Jiffry M.Z., Ahmed-Khan M.A. (2023). Evaluating Dental Fear and Anxiety in Pediatric Patients Visiting a Private and a Public Dental Hospital in Lahore, Pakistan. Cureus.

[44] Muglali M., Komerik N. (2008). Factors related to patients’ anxiety before and after oral surgery. J. Oral Maxillofac. Surg..

[45] López-Jornet P., Camacho-Alonso F., Sanchez-Siles M. (2014). Assessment of general pre and postoperative anxiety in patients undergoing tooth extraction: A prospective study. Br. J. Oral Maxillofac. Surg..

[46] Vello-Ribes M.A., Aura-Tormos J.I., Valero-Contelles C., Casaña-Ruiz M.D., Catala-Pizarro M. (2026). Behavioural Impact of Parental Presence Versus Absence in Paediatric Dentistry: A Systematic Review. Dent. J..

[47] Alsadat F.A., El-Housseiny A.A., Alamoudi N.M., Elderwi D.A., Ainosa A.M., Dardeer F.M. (2018). Dental fear in primary school children and its relation to dental caries. Niger. J. Clin. Pract..

[48] Wogelius P., Poulsen S., Sørensen H.T. (2003). Prevalence of dental anxiety and behavior management problems among six to eight years old Danish children. Acta Odontol. Scand..

[49] Passos De Luca M., Massignan C., Bolan M., Butini Oliveira L., Aydinoz S., Dick B., De Luca Canto G. (2021). Does the presence of parents in the dental operatory room influence children’s behaviour, anxiety and fear during their dental treatment? A systematic review. Int. J. Paediatr. Dent..

[50] Katz M.H. (2003). Multivariable analysis: A primer for readers of medical research. Ann. Intern Med..

[51] Foley M., Akers H.F. (2019). Does poverty cause dental caries?. Aust. Dent. J..

